# A Post-Burst Afterdepolarization Is Mediated by Group I Metabotropic Glutamate Receptor-Dependent Upregulation of Ca_v_2.3 R-Type Calcium Channels in CA1 Pyramidal Neurons

**DOI:** 10.1371/journal.pbio.1000534

**Published:** 2010-11-16

**Authors:** Jin-Yong Park, Stefan Remy, Juan Varela, Donald C. Cooper, Sungkwon Chung, Ho-Won Kang, Jung-Ha Lee, Nelson Spruston

**Affiliations:** 1 Department of Neurobiology and Physiology, Northwestern University, Evanston, Illinois, United States of America; 2 Department of Life Science and Basic Science Institute for Cell Damage Control, Sogang University, Seoul, Korea; European Brain Research Institute, Italy

## Abstract

Activation of group I metabotropic glutamate receptors (subtypes mGluR1 and mGluR5) regulates neural activity in a variety of ways. In CA1 pyramidal neurons, activation of group I mGluRs eliminates the post-burst afterhyperpolarization (AHP) and produces an afterdepolarization (ADP) in its place. Here we show that upregulation of Ca_v_2.3 R-type calcium channels is responsible for a component of the ADP lasting several hundred milliseconds. This medium-duration ADP is rapidly and reversibly induced by activation of mGluR5 and requires activation of phospholipase C (PLC) and release of calcium from internal stores. Effects of mGluR activation on subthreshold membrane potential changes are negligible but are large following action potential firing. Furthermore, the medium ADP exhibits a biphasic activity dependence consisting of short-term facilitation and longer-term inhibition. These findings suggest that mGluRs may dramatically alter the firing of CA1 pyramidal neurons via a complex, activity-dependent modulation of Ca_v_2.3 R-type channels that are activated during spiking at physiologically relevant rates and patterns.

## Abstract 2

The excitability of hippocampal pyramidal neurons is regulated by activation of metabotropic glutamate receptors, an effect that is mediated by modulation of R-type calcium channels.

## Author Summary

The hippocampus is an essential structure in the brain for the formation of new declarative memories. Understanding the cellular basis of memory formation, storage, and recall in the hippocampus requires a knowledge of the properties of the relevant neurons and how they are modulated by activity in the neural circuit. For many years, we have known that various chemical neurotransmitters can modulate the electrical excitability of neurons in the hippocampus. Here, we report new experiments to reveal how the chemical neurotransmitter glutamate increases neuronal excitability. The effect we study is the conversion of the afterhyperpolarization (a cellular consequence of firing an action potential) to an afterdepolarization. We identified the metabotropic glutamate receptors involved in this conversion (receptors called mGluR1 and mGluR5) as well as the final target of modulation (R-type calcium channels composed of Ca_v_2.3 subunits), which cause the neurons to exhibit altered excitability in the presence of glutamate. We also determined some of the intermediate steps between activation of the glutamate receptors and modulation of the calcium channels responsible for the change in excitability, offering further mechanistic insight into how synaptic transmission can regulate cellular and network activity.

## Introduction

Metabotropic glutamate receptors (mGluRs) are a class of G-protein coupled receptors that may mediate a variety of effects through presynaptic and postsynaptic actions. Because these receptors are activated by glutamatergic neurons during network activity, they are in a position to regulate neural function in an activity-dependent manner. The effects of mGluR activation may be rapid or long-lasting, so they are important for short-term and long-term regulation of neural activity [1]. They have been implicated in physiological functions, such as learning [2]–[5], as well as in a number of neurological disorders [6], including mental retardation, epilepsy, and Alzheimer's disease [7]–[11].

Among the many effects of mGluR activation, the modulation of neuronal excitability has a direct effect on the response of cortical pyramidal neurons to excitatory synaptic input. The effects of mGluRs on excitability are commonly mediated by group I mGluRs, resulting in modulation of voltage-gated Na^+^, Ca^2+^, or K^+^ currents, as well as Ca^2+^-activated K^+^ currents, nonselective cation currents, or ion exchanger currents [1]. Modulation of these targets by group I mGluRs typically increases postsynaptic excitability [12],[13]. Thus, group I mGluRs may modulate network function through modulation of multiple ion channels, resulting in enhanced excitability of glutamatergic pyramidal neurons.

Activation of group I mGluRs in cortical and hippocampal pyramidal neurons has been reported to reduce the post-spike AHP and induce an ADP [14]–[18], but the receptors, signal transduction mechanisms, and ion channels responsible for this effect are incompletely understood. Because of the importance of these modulatory effects for hippocampus-dependent functions and diseases, we studied the effects of activating group I mGluRs on the excitability of hippocampal CA1 pyramidal neurons. We report here that activation of these receptors results in enhanced activity of Ca_v_2.3 R-type calcium channels, thus producing a medium ADP lasting a few hundred milliseconds. A slow ADP lasting for seconds is mediated by different mechanisms.

## Results

### Activation of Group I mGluRs Induces an Afterdepolarization

**Figure 1:**
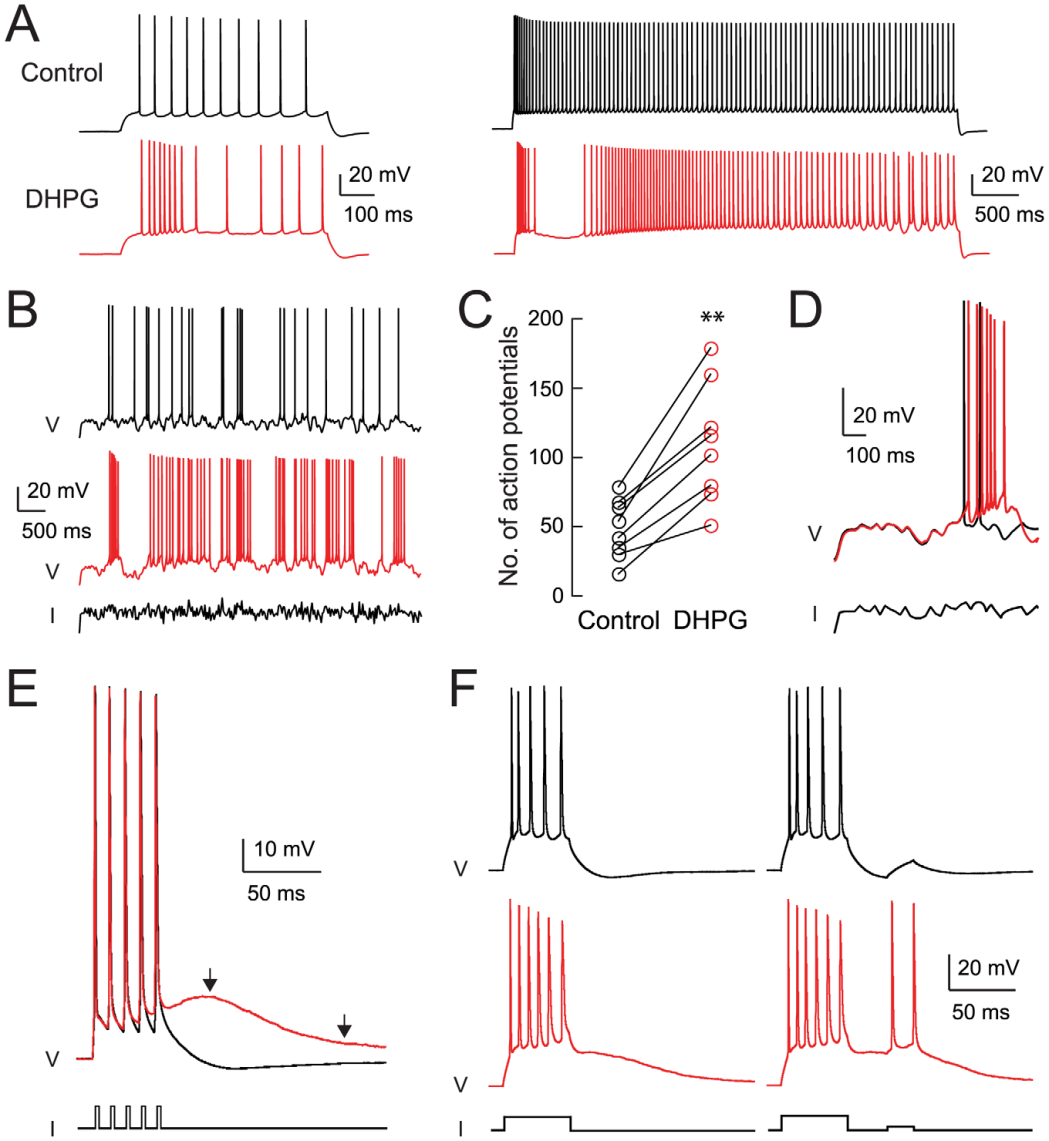
Activation of group I mGluRs enhances excitability of CA1 pyramidal neurons. (A) Responses to step current injections of 300 pA for 0.6 s (left) or 400 pA for 4.5 s (right). Black traces are control responses in normal ACSF, just before the onset of DHPG perfusion (t = 0 min). Red traces are after 15 min (t = 15 min) of application of 2 µM DHPG (see Materials and Methods). (B) Responses to noisy current injection (200 pA step plus noise for 10 s; first 6 s shown). (C) Number of action potentials evoked by 10-s long, noisy current injections before (t = 0 min) and after (t = 15 min) application of DHPG (paired *t* test, *p*<0.001). (D) Expanded plot of the initial portion of response shown in (B). Note the lack of effect of DHPG on subthreshold responses prior to the first spike. (E) Response to five brief current injections (2 nA, 2 ms each) to evoke a burst of action potentials in control (t = 0 min) or DHPG (t = 15 min). First arrow indicates peak of the post-burst ADP in DHPG and second arrow indicates time of decay to 25% of peak. (F) Responses to step current injections (800 pA, 50 ms) to evoke a burst of action potentials, with and without a second step (200 pA, 20 ms) to test for excitability during the post-burst ADP.

We obtained whole-cell current-clamp recordings from CA1 pyramidal neurons in rat hippocampal slices. The effects of the group I mGluR activation on responses to intracellular current injection were examined following bath application of the group I mGluR agonist DHPG (2–4 µM, see Materials and Methods). Step current injections (0.6-s long) that were just above threshold for action potential firing in control elicited increased action potential firing in the presence of DHPG (Figure 1A). In response to longer current injections (4.5 s), DHPG converted a simple pattern of spike-frequency accommodation to a more complex pattern consisting of a high-frequency burst of action potentials, followed by a period of silence, and finally a continuous train exhibiting spike-frequency accommodation. In response to noisy current injections, the number of action potentials was also increased when DHPG was applied (Figure 1B and 1C). DHPG produced a small but statistically significant increase in the input resistance (54.7±3.0 MΩ for control, t = 0 min; 59.7±4.1 MΩ for DHPG, t = 15 min; *n* = 26, paired *t* test, *p*<0.01). This did not noticeably change the subthreshold response to noisy current injection, but enhanced spiking appeared to be attributable to a reduction in the post-spike AHP in the presence of DHPG (Figure 1D).

The effect of DHPG on the post-spike AHP was studied systematically by examining responses to bursts of action potentials evoked by five brief (2 nA, 2 ms) current injections. In normal artificial cerebrospinal fluid (ACSF), a 100 Hz burst of five spikes was followed by an AHP (−3.1±0.2 mV) that reached a peak at 59±5 ms after the last spike. Application of DHPG (or quisqualate, another group I mGluR agonist, Figure S1) eliminated the AHP, resulting in a post-burst ADP (+18.7±0.7 mV) that reached a peak at 34±3 ms after the last spike and decayed to 25% of the peak value in 200±23 ms (*n* = 20, Figure 1E). We refer to this ADP as a medium ADP to distinguish it from the fast ADP following a single spike in normal ACSF [19]–[23] and a slow DHPG-induced ADP described later in this article. Functionally, the change from post-burst AHP into a post-burst medium ADP made the pyramidal neurons more excitable; current injections that were subthreshold during the post-burst AHP evoked action potential firing during the medium ADP (Figure 1F).

Application of DHPG resulted in a gradual reduction of the AHP and conversion to an ADP. The medium ADP reached its maximum value after several minutes in DHPG and was fully reversible, with a similar time course, upon washout of DHPG (Figure S2); however, the slow onset and reversal of the medium ADP is attributable to the slow speed of the perfusion system, as rapid application of DHPG produced more rapid responses (see below). In all experiments, the amplitude of the post-burst potential (AHP or medium ADP) was quantified at a fixed time, corresponding to the peak of the AHP in normal ACSF (59±5 ms after the last spike). For some analyses, the effect of DHPG was quantified by the change in the post-burst potential (Δ post-burst potential) at this time point in the response (see Figure S3). This measure required comparison of the response in normal ACSF (AHP at t = 0 min) to the response at a fixed time after application of DHPG (e.g., ADP at t = 15 min). Importantly, this measure was not complicated by any slow, drug-independent effects, as the post-burst AHP was stable for typical recording duration in normal ACSF (Figure S3).

**Figure 2:**
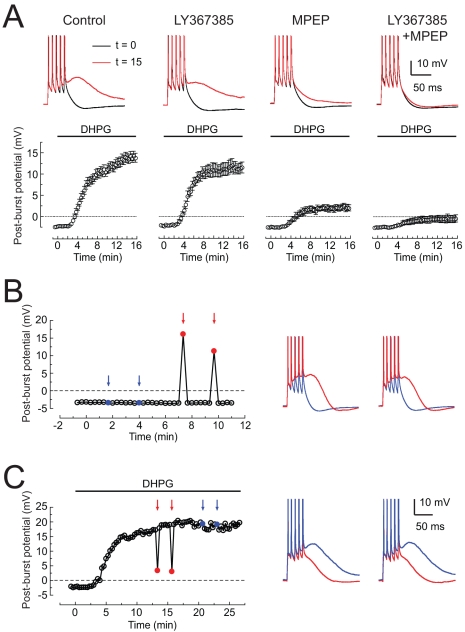
Metabotropic glutamate receptors mediating the DHPG-induced ADP. (A) Top: sample responses to bursts of five action potentials in normal ACSF (black) or following application of 2.5 µM DHPG (red) in four different conditions. Drug concentrations are 25 µM LY367385 and 10 µM MPEP. Bottom: time course of medium ADP amplitude (58 ms after last spike) following application of 2–4 µM DHPG beginning at t = 0 min. (B) Post-burst potential plotted over time in normal ACSF. On select trials, 500 µM DHPG was applied locally, via pressure application from a broken patch pipette (see Materials and Methods) approximately 3 s before the current-injection stimulus. Blue arrows and traces indicate application to the distal apical dendrites (near the border of stratum radiatum and stratum lacunosum-moleculare); red arrows and traces indicate application to the perisomatic region (stratum pyramidale). (C) Same as (B) except 2–4 µM DHPG was applied to the bath beginning at t = 0 min and normal ACSF was applied using localized pressure application to the perisomatic (red) or apical dendritic (blue) regions. In all panels, the stimulus is five brief current injections and the interval between trials is 20 s. Action potentials are truncated.

To determine which group I mGluR subtype was responsible for the DHPG-induced medium ADP, we used the subtype-selective antagonists LY367385 (25 µM, mGluR1 antagonist) and MPEP (10 µM, mGluR5 antagonist). LY367385 blocked the medium ADP (t = 15 min) by 16%, while MPEP blocked it by 73% (Figure 2A), suggesting that the effect of DHPG is mediated primarily by mGluR5 and partially by mGluR1. When applied together, the two drugs blocked the medium ADP by 88% (Figure 2A), suggesting that the effects of mGluR1 and mGluR5 are approximately additive. To determine the subcellular localization of the mGluRs mediating the medium ADP, DHPG was applied locally, by pressure application from a large patch pipette, to either the perisomatic region or the apical dendrites (see Materials and Methods for details). Application of DHPG (500 µM in application pipette) to the apical dendrites had no effect, while perisomatic application produced a medium ADP (Figure 2B). Similarly, when DHPG was present in the bath, application of normal ACSF reduced the medium ADP when applied to the soma, but not when applied to the apical dendrites (Figure 2C). The effects of DHPG were induced within seconds of its application and reversed rapidly when the DHPG application was terminated. Together, these results suggest that perisomatic mGluRs must be activated to elicit an medium ADP in response to somatic action potential firing and that the effect is rapidly induced and reversed. The lack of effect with application to the dendrites does not necessarily imply the absence of group I mGluRs, as backpropagating action potentials could have a different effect from action potentials in the soma (see Discussion).

**Figure 3:**
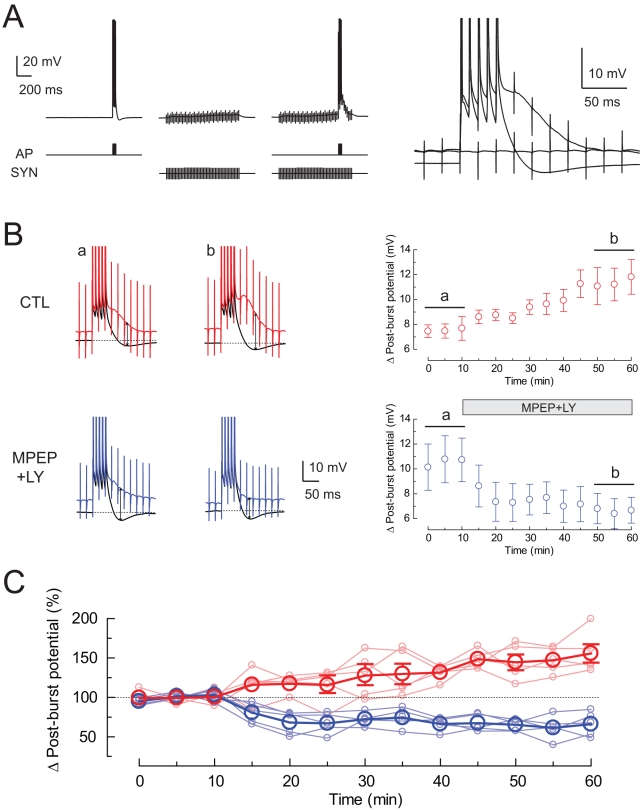
Synaptic stimulation during somatic action potentials induces a post-burst ADP. (A) Synaptic stimulation (SYN) was paired with somatic action potentials (AP) to evoke the post-burst ADP. Stimuli were 100 Hz five action potentials only, 50 Hz for 1 s of synaptic stimulation only, or both stimuli combined together. Examples for each condition from a single cell are overlaid on the right. (B) Time course of the change in post-burst potential in control and group I mGluR receptor antagonists. Representative average responses were obtained from three consecutive traces in control and MPEP/LY. Arrows indicate the amplitude of the change in post-burst potentials. The baseline membrane potential is indicated by dotted lines. (C) Normalized change in post-burst potential in control and MPEP/LY (*n* = 5 cells in each condition; results for each cell are shown by the lightly colored lines).

To determine whether group I mGluR-mediated modulation of the post-burst potential could occur in response to synaptically released glutamate, the post-burst AHP/ADP was compared during control conditions and during high-frequency (50 Hz) activation of Schaffer collaterals. Fast synaptic responses were prevented by blocking glutamate and GABA receptors (see Materials and Methods). Under control conditions, each burst was followed by an AHP. During synaptic stimulation, however, each burst was followed instead by an ADP (Figure 3A). To determine the role of group I mGluR activation in the induction of the post-burst ADP, we applied blockers of mGluR1 and mGluR5 (Figure 3B and 3C). The results of these experiments were compared to a separate group of control experiments occurring over the same time course but without blocker application. In the absence of synaptic stimulation, the post-burst AHP was stable over time, both in control and in the presence of group I mGluR blockers (Figure S4). In the presence of synaptic stimulation, the size of the ADP in the control group gradually increased over the course of the experiment (156%±12% of initial value, *n* = 5). By contrast, blocking the group I mGluRs resulted in a decrease in the size of the ADP (66%±7% of initial value, *n* = 5). The magnitude of the ADP at the end of the experiment (60 min) in the presence of group I mGluR blockers was 42% of control, consistent with a substantial contribution of these receptors to induction of the ADP triggered by synaptically released glutamate. The long-term effects of synaptic stimulation on the ADP are interesting but are not considered here. Instead, our focus is on the acute modulation of the AHP/ADP during activation of group I mGluRs.

Bath application of DHPG increased the fast ADP following a single spike, and the size of the medium ADP increased with both the number and the frequency of action potentials, reaching medium ADP values of nearly 15 mV for 5 spikes at 100 Hz (Figure S3). Longer trains (20 or 50 spikes) did not increase the ADP further and in fact resulted in a decrease in the size of the ADP (Figure S5), perhaps due to inactivation of the ADP-producing current or enhanced activation of an AHP-producing current.

**Figure 4:**
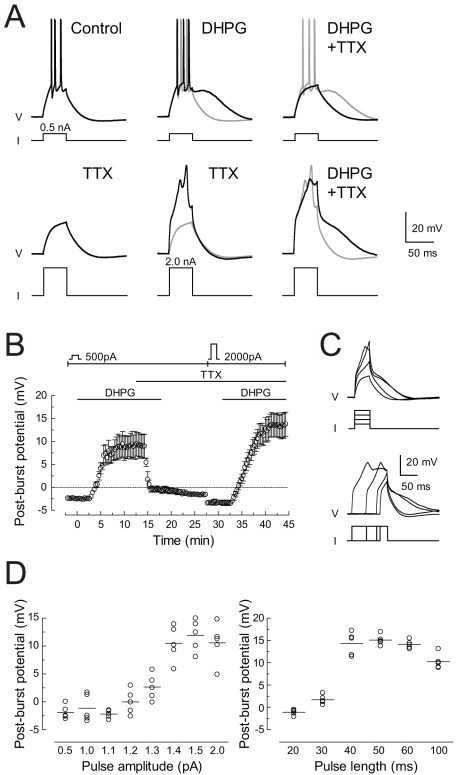
The post-burst ADP induced by DHPG requires action potential firing or a calcium spike. (A) Responses to step current injections (0.5 or 2.0 nA, 40 ms) in various conditions, as indicated. Note the calcium spike in response to the larger current injection in the presence of TTX (0.5 µM). Action potentials are truncated in the first two traces. Gray traces are superimpositions of the previous response. (B) Summary of experiments like the example shown in (A). Interval between trials is 20 s. (C) Effects of step current amplitude (top: 0.5, 1.0, 1.5, 2.0 nA for 40 ms) and duration (bottom: 1.5 nA for 20, 30, 60, 100 ms) on calcium spikes and the resultant ADP. (D) Summary of experiments like the example shown in (C).

The medium ADP following a burst of spikes evoked by a step current injection was blocked by application of tetrodotoxin (TTX; 0.5 µM) to eliminate Na^+^-mediated spikes; however, increasing the magnitude of the current injection to elicit a Ca^2+^ spike [24] restored the medium ADP (Figure 4A and 4B). Under these conditions, the amplitude of the medium ADP increased with the magnitude and duration of the current injection, reaching a maximum of about 10–15 mV for current injections of at least 1.4 nA for 40 ms (Figure 4C and 4D).

### The DHPG-Induced Medium ADP Requires Activation of Voltage-Gated Calcium Channels

**Figure 5:**
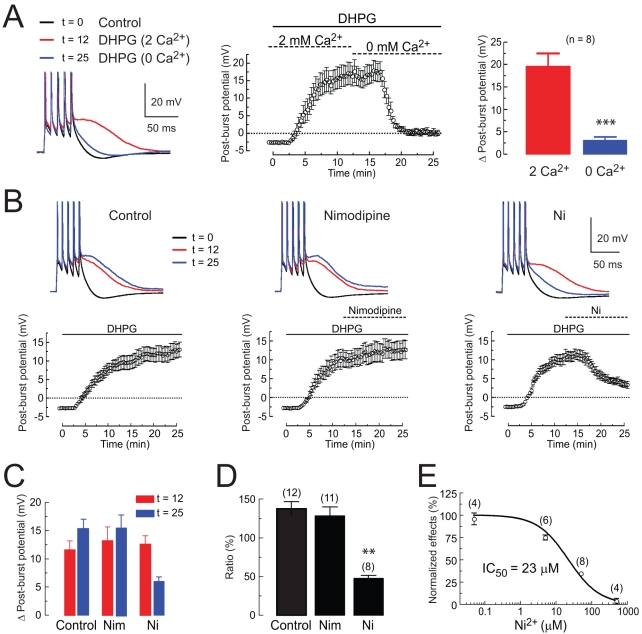
The post-burst ADP induced by DHPG requires extracellular calcium entry. (A) Responses in control (t = 0 min, black), ACSF with 2–4 µM DHPG and 2 mM Ca^2+^ (red) and ACSF with 2–4 µM DHPG and zero Ca^2+^ (blue). Time course of the experiment and summary data are also shown (*** paired *t* test, *p*<0.0001). (B) Sample responses and time courses for experiments to test effects of nimodipine (10 µM) and NiCl_2_ (Ni, 50 µM) on the DHPG-induced post-burst ADP. (C) Summary of effects of nimodipine (Nim) and NiCl_2_ (Ni) in DHPG alone (2–4 µM; *t* = 12 min) and DHPG plus drug (*t* = 25 min). (D) Ratio of post-burst ADP at *t* = 25 min to ADP at *t* = 12 min. Note that this ratio is greater than one in control, because DHPG increases the amplitude of the post-burst ADP over this time. One-way ANOVA, *p*<0.0001, with post hoc tests versus control: ** *p*<0.001. (E) Concentration dependence of the block of the post-burst ADP by Ni^2+^. Values are normalized to the control ratio in (D).

The requirement for action potential firing or a Ca^2+^ spike suggests that the medium ADP may require Ca^2+^ entry through voltage-gated calcium channels (VGCCs). Consistent with this idea, we found that the medium ADP was eliminated by switching to a Ca^2+^-free ACSF (Figure 5A) or by bath application of micromolar concentrations of NiCl_2_ (Figure 5B–E; IC_50_ = 23 µM). Nimodipine (10 µM, an L-type calcium channel blocker) did not block the medium ADP (Figure 5B–D). To test whether the medium ADP required elevation of internal Ca^2+^ concentration, we performed experiments with patch-clamp electrodes containing BAPTA (10 mM) and found that this strongly reduced the medium ADP (Figure S6). Blocking Ca^2+^ release from internal stores with cyclopiazonic acid (CPA; 20 µM) also reduced the medium ADP (Figure S6), suggesting that this is another important mechanism for induction of the medium ADP.

**Figure 6:**
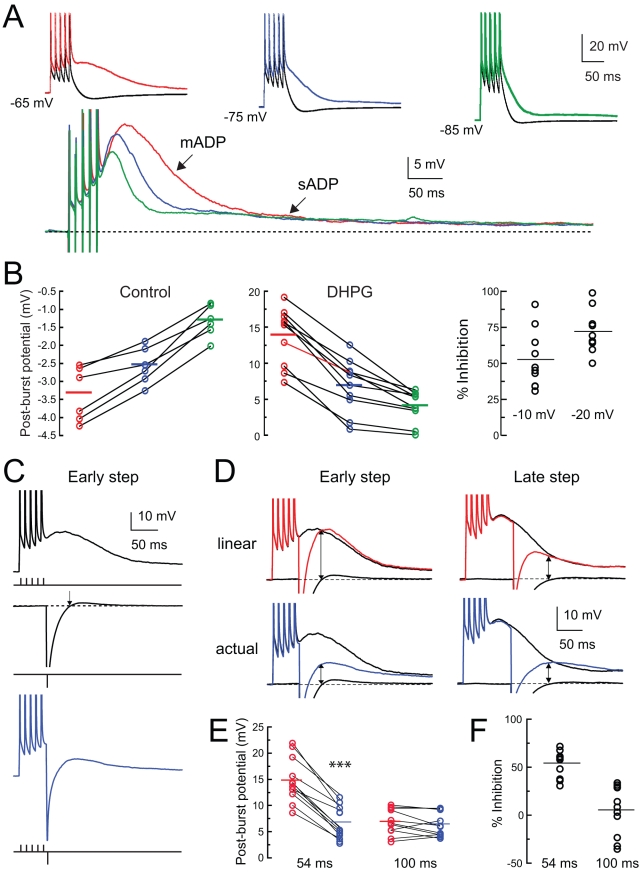
The post-burst ADP induced by DHPG is voltage dependent. (A) Responses in normal ACSF (control, black) and 2.5 µM DHPG at holding potentials of −65, −75, and −85 mV (red, blue, and green). Below are subtractions of the control response from the DHPG response for each pair, clearly revealing a voltage-dependent medium ADP (mADP) and a voltage-independent slow ADP (sADP). Stimulus is five brief current stimuli (as in Figure 1E) and action potentials are truncated. (B) Summary of experiments like the one shown in (A). Hyperpolarization decreased the post-burst AHP in control and decreased the post-burst medium ADP in DHPG (repeated-measures one-way ANOVA, *p*<0.0001 for effect of holding potential in both control and DHPG). Holding potentials were −65.4±0.2 mV, −75.4±0.2 mV, −85.8±0.2 mV in control (*n* = 6) and −65.9±0.2 mV, −75.6±0.2 mV, −85.6±0.2 mV in DHPG (*n* = 10). Inhibition of the medium ADP by hyperpolarization, plotted as a percentage of the medium ADP amplitude (right). (C) Responses in 2.5 µM DHPG to three stimuli: five brief depolarizing current injections (black), one hyperpolarizing current injection (black, truncated, returned to baseline at arrow), or both combined (blue). (D) Top: the post-burst ADP in DHPG is compared to the linear sum (red) of the post-burst ADP and the hyperpolarizing response. Bottom: the post-burst ADP is compared to the combined response (blue). The hyperpolarizing response was delivered either 8 ms (early step) or 48 ms (late step) after the last action potential. The amplitude of the response (arrows) was measured at the time when the hyperpolarizing response decayed back to rest, which was 54 ms (early step) or 100 ms (late step) after the last action potential. Action potentials are truncated. (E) Comparison of the expected response (from linear sum, red) and the actual response amplitude (blue) for the early step (54 ms) and late step (100 ms). Paired *t* test: *** *p*<0.0001 for 54 ms, *p* = 0.18 for 100 ms. (F) Inhibition of the medium ADP by the early and late hyperpolarization, plotted as a percentage of the medium ADP amplitude.

The requirement for Ca^2+^ entry through VGCCs and the elevation of internal Ca^2+^ concentration is consistent with two different models of the medium ADP. In the first model, the medium ADP is mediated by Ni^2+^-sensitive VGCCs, with their modulation (enhanced activity) by DHPG requiring elevated intracellular Ca^2+^. In the second model, Ca^2+^ entry through VGCCs contributes little to the medium ADP directly but acts as a trigger for a downstream conductance that is modulated by DHPG. For example, Ca^2+^ entry and release from internal stores could activate nonselective cation currents, such as I_CAN_, which mediate the medium ADP. Alternatively (or in addition), downregulation of Ca^2+^-activated K^+^ channels by DHPG could unveil the medium ADP. To distinguish between these possibilities, we examined the voltage dependence of the medium ADP. We reasoned that in the first model, where the medium ADP is mediated by VGCCs directly, hyperpolarization should accelerate deactivation of the VGCCs, thus reducing the medium ADP. In the second model, hyperpolarization would not eliminate Ca^2+^ entry during the action potentials, and it would increase the driving force on the cation channels, thus increasing the medium ADP. We found that holding the cell at a hyperpolarized holding potential strongly reduced the amplitude and duration of the medium ADP (Figure 6A and 6B), a finding most consistent with the first model, in which the medium ADP is mediated by VGCCs directly. However, the slowest component of the medium ADP was not reduced by hyperpolarization (Figure 6A), suggesting a contribution of Ca^2+^-activated channels to a slow ADP (see below).

To further examine the voltage dependence of the medium ADP, we delivered short hyperpolarizing current injections (−6 nA, 2 ms) either 8 or 48 ms after the last action potential in a burst (Figure 6C). On its own, the brief current injections produced a hyperpolarization to about −120 mV (see Materials and Methods) and returned to rest in 46±2 ms (*n* = 12). Hyperpolarization at the early time point reduced the medium ADP amplitude more than expected by simply summing the medium ADP and the short hyperpolarization alone (Figure 6D and 6E), again consistent with the notion that the medium ADP is mediated by a voltage-dependent conductance that can be deactivated by hyperpolarization. This effect was measured at the time when the hyperpolarizing response on its own decayed back to rest (54±2 ms after the last spike). When the hyperpolarizing current step began 48 ms after the last action potential, its effect (again measured when the hyperpolarization on its own decayed to rest; 100±2 ms after the last spike) was not statistically significant (Figure 6D and 6E). This finding suggests that the VGCCs responsible for the medium ADP remain activated for at least 50 ms, but not longer than 100 ms, while the conductance responsible for the slow ADP is not deactivated by hyperpolarization.

**Figure 7:**
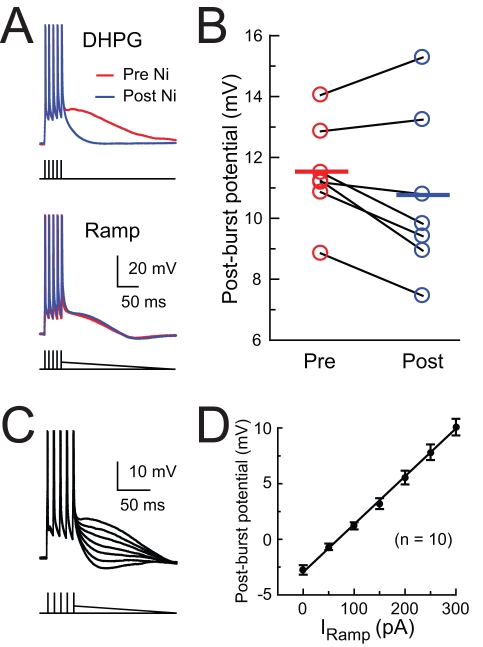
Post-burst depolarization only activates Ni^2+^-sensitive Ca^2+^ channels during mGluR activation. (A) The DHPG-induced medium ADP is blocked by application of 500 µM Ni^2+^, but an artificial medium ADP produced by a ramp current injection after the spikes is unaffected by Ni^2+^. (B) Summary of Ni^2+^ effects on ramp-induced medium ADP. Paired *t* test, *p* = 0.14. (C) A variable ramp current produced a linearly increasing medium ADP. (D) Summary plot showing a linear effect of the ramp-current amplitude (I_Ramp_, initial amplitude) on post-burst potential.

The voltage dependence of the medium ADP, combined with its sensitivity to low concentrations of Ni^2+^, suggests that the medium ADP may result from upregulation of Ca_v_2.3 R-type calcium channels. However, previous work has indicated that mGluR activation can downregulate K^+^ channels [17],[18],[25]–[28], which could result in inhibition of the AHP and activation of R-type channels, without any actual modulation of the calcium channels by mGluRs. To test this alternative hypothesis, we converted the medium AHP to a medium ADP by injecting a ramp current that followed action potential firing, resulting in an artificial medium ADP in control ACSF, which resembled the medium ADP following application of DHPG. This artificial medium ADP was unaffected by bath application of Ni^2+^, suggesting that the Ni^2+^-sensitive calcium channels are not significantly activated by a post-burst medium ADP in the absence of mGluR activation (Figure 7A and 7B). We also tested the voltage dependence of the medium ADP in the absence of DHPG by varying the initial amplitude of the ramp current. The relationship between the medium ADP amplitude and the current injection was linear (Figure 7C and 7D), suggesting that voltage-dependent conductances do not amplify the medium ADP over this voltage range in the absence of mGluR activation.

**Figure 8:**
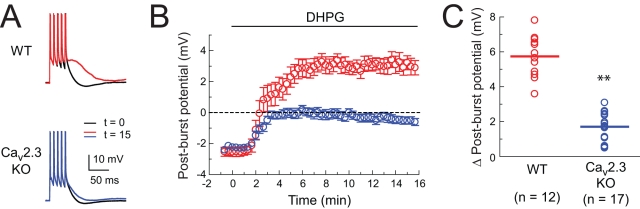
Ca_v_2.3 knockout mice exhibit a reduced DHPG-induced medium ADP compared to wild-type mice. (A) Examples of responses in control (black) and following application of 10 µM effect of DHPG (red, blue) in wild-type (WT) and Ca_v_2.3 knockout (KO) mice. Stimulus is five brief current stimuli (as in Figure 1E). Action potentials are truncated. (B) Time course of effects of DHPG in WT (red) and Ca_v_2.3 KO (blue) mice. Interval between trials is 20 s. (C) Summary of effects of DHPG on medium ADP in WT (red) and Ca_v_2.3 KO (blue) mice. Unpaired *t* test: ** *p*<0.001.

To further test the hypothesis that upregulation of R-type calcium channels is responsible for the DHPG-induced medium ADP, we performed experiments on Ca_v_2.3 knockout mice [29]. The medium ADP induced by DHPG was significantly smaller in the knockout mice, compared to wild-type controls (Figure 8). Because the Ca_v_2.3 knockout mice are a hybrid of C57BL/6J (black) and 129S1/SvImJ (brown) mice, pups had different coat colors (black, dark brown, light brown; see Materials and Methods). We analyzed the results from different-colored mice separately and found no differences between these groups. Furthermore, we performed control experiments using black and brown mice and found similar DHPG-induced medium ADP amplitude in each of the wild-type strains (Figure S7).

**Figure 9:**
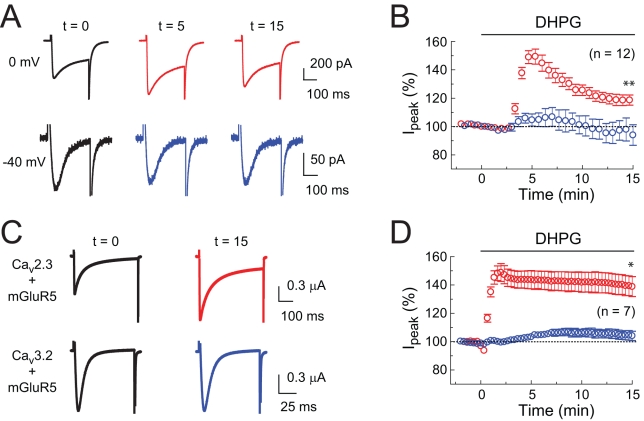
Voltage-clamp analysis of voltage-gated calcium channel modulation by DHPG. (A) Voltage-clamped Ca^2+^ currents in CA1 pyramidal neurons. DHPG enhances currents evoked by steps from −70 to 0 mV (5 µM DHPG, red; control, black), but not to −40 mV (5 µM DHPG, blue; control, black). Times after application of DHPG are indicated in minutes. (B) Summary of the time course of effects of DHPG on currents in CA1 pyramidal neurons. Paired *t* test at t = 15 min, ** *p*<0.001 versus t = 0 min for steps to 0 mV and *p* = 0.63 for steps to −40 mV. Interval between trials is 20 s. (C) Voltage-clamped Ba^2+^ currents (steps from −90 to 0 mV) in oocytes expressing mGluR5 together with either Ca_v_2.3 (5 µM DHPG, red; control, black) or Ca_v_3.2. (5 µM DHPG, blue; control, black). (D) Summary of the time course of effects of DHPG on Ba^2+^ currents in oocytes. Interval between trials is 20 s. DHPG enhances currents in oocytes expressing Ca_v_2.3, but not Ca_v_3.2. Paired *t* test at t = 15 min, * *p*<0.01 versus t = 0 min for Ca_v_2.3 and *p* = 0.27 for Ca_v_3.2.

We also used voltage-clamp recording to measure isolated R-type calcium currents in CA1 pyramidal neurons (see Materials and Methods). The resulting currents were upregulated by DHPG when activated by large but not small depolarizing steps (Figure 9A and 9B), consistent with modulation of R-type (high voltage activated) but not T-type (low voltage activated) calcium currents. Finally, to explore the ability of mGluR5 activation to regulate current mediated by calcium channels, we co-expressed, in *Xenopus* oocytes, mGluR5 along with either Ca_v_2.3 α_1_ (plus α_2_δ_1_ and β_3_) or Ca_v_3.2 α_1_ (see Materials and Methods; [30]). We found that DHPG application upregulated barium currents in oocytes expressing mGluR5 and Ca_v_2.3 but not mGluR5 and Ca_v_3.2, suggesting subunit selective modulation of R-type, but not T-type, calcium channels by mGluR5 activation with DHPG (Figure 9C and 9D).

The observation that the slow ADP was not eliminated by hyperpolarization suggests that a different class of channels may contribute to the slow ADP. We therefore examined this component of the ADP pharmacologically. It was not blocked by NiCl_2_ (Figure S8A and S8B), consistent with the idea that it is not mediated by the VGCCs responsible for the medium ADP. The slow ADP was also unaffected by nimodipine (Figure S8A and S8B).

We performed a battery of pharmacological experiments to explore the signal transduction mechanisms responsible for the post-burst medium ADP and slow ADP. These experiments (Figure S8C and S8D) required either intracellular drug application or pre-incubation of the drug in the bath. Like the medium ADP, the slow ADP was blocked by group I mGluR antagonists, especially the mGluR5 blocker MPEP. The slow ADP was also reduced by intracellular BAPTA, but it was not blocked by drugs that interfere with Ca^2+^ release from intracellular stores (CPA, ruthenium red, or heparin). All of these drugs inhibited the medium ADP, suggesting the Ca^2+^ release is required for the medium ADP, but not the slow ADP. The medium ADP was blocked by intracellular GDP-β-S, which interferes with G-protein coupled signaling, or by the PLC inhibitor U73122 (but not the inactive analog U73343). None of these drugs blocked the slow ADP, however, suggesting further that distinct signaling mechanisms mediate the DHPG-induced medium ADP and slow ADP.

### Activity-Dependence of the DHPG-Induced Medium ADP

**Figure 10:**
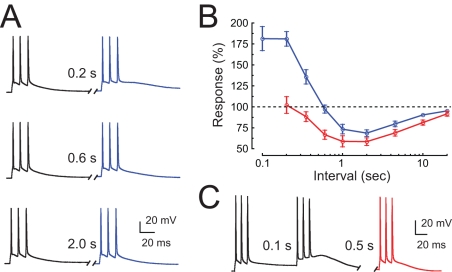
Activity dependence of the post-burst medium ADP in the presence of DHPG. (A) Responses to pairs of bursts delivered at different intervals after 20 min application DHPG (2.5 µM). Stimulus for each burst is three brief current stimuli (2 nA, 2 ms). (B) Plot of amplitude of the second medium ADP to the first medium ADP (blue) or the amplitude of the third medium ADP to the first medium ADP (red) as a function of the interval between the two bursts. The medium ADP amplitude was measured at 28 ms after last spike in each burst. (C) Response for the three-burst experiment, where the interval between the first and second burst was 0.1 s and the interval between the second and third burst was 0.5 s (corresponding to the point at 0.6 s in B).

We examined the activity dependence of the DHPG-induced post-burst medium ADP by delivering pairs of bursts at intervals of 0.1 to 20 s. Three-spike bursts were used in order to limit the size of the medium ADP so that either facilitation or inhibition could be observed. At intervals up to 200 ms, the second burst evoked a medium ADP almost twice the size of the first; at intervals of 1–5 s, the second burst was reduced by about 25% (Figure 10). The data were well fit by a model consisting of two processes: a facilitation process with a decay time constant of 0.25 s and an inhibition process with a decay time constant of 10 s (Figure S9 and Text S1). In the model, inhibition affected the fraction of the current available to be activated and facilitation affected the probability of activation (by a burst) of the available current. A key feature of the model was that any portion of the current could be inhibited, regardless of whether or not it was activated. This model predicted that facilitation is not expected, but inhibition persists, for a third burst delivered following two bursts (Figure S9). We conducted this experiment and the results were consistent with the predictions of the model (Figure 10). By contrast, a model in which inactivation was limited to the activated channels could not explain the results of the three-pulse experiment (Figure S9 and Text S1).

## Discussion

The findings reported here suggest that activation of group I mGluRs, which can occur as a result of synaptically released glutamate, increases the excitability of CA1 pyramidal neurons primarily by converting the post-spike AHP to an ADP via group I mGluR-mediated upregulation of Ca_v_2.3 R-type calcium channels. The largest component of this change is a medium ADP lasting a little over 200 ms. A longer-lasting slow ADP (seconds) was smaller and mediated by different ion channels and signal transduction pathways than the medium ADP.

The medium ADP required action potential firing, although calcium spikes also activated the medium ADP in DHPG. The medium ADP was not affected by blocking L-type VGCCs with nimodipine, but calcium entry through Ni^2+^-sensitive channels was required for the medium ADP, as was intracellular Ca^2+^ elevation and Ca^2+^ release from internal stores. Block of the medium ADP by micromolar Ni^2+^ and the strong reduction of mGluR-mediated modulation of the post-burst potential in Ca_v_2.3 knockout mice suggest that activation of R-type VGCCs are required for conversion of the medium AHP to a medium ADP [31]–[33]. Although it is difficult to distinguish between direct and indirect contributions of these channels, the voltage sensitivity of the medium ADP—including inhibition of the medium ADP by hyperpolarization after the triggering action potentials—suggests that R-type VGCCs contribute directly to the medium ADP. Our voltage-clamp experiments suggest the existence of an R-type Ca^2+^ current even prior to activation of mGluRs. The presence of an AHP under control conditions suggests, however, that K^+^ currents are larger than Ca^2+^ currents. Activation of mGluRs may downregulate K^+^ currents, but this downregulation is not sufficient to explain a voltage-dependent ADP as indicated by the ramp current experiments (Figure 7), which show that upregulation of R-type Ca^2+^ current is required to produce the ADP.

We cannot rule out the possibility that Ca^2+^ entry activates a voltage-dependent cation current underlying the medium ADP. Indeed, a number of reports implicate the activation of cation currents following group I mGluR activation in hippocampal neurons [34]–[39]. Some of these currents are Ca^2+^ sensitive, some are voltage sensitive, and others are both Ca^2+^ and voltage sensitive. Expression of these currents varies between CA3 and CA1 pyramidal neurons [40]. All of these currents are slower than the medium ADP reported here. Furthermore, none of these currents have been reported as sensitive to micromolar concentrations of Ni^2+^. Thus, the most parsimonious explanation of our data is that DHPG upregulates Ni^2+^-sensitive R-type VGCCs, which remain active for about 100 ms following a burst of spikes.

At least some of the slow, group I mGluR-activated currents described previously may be responsible for the slow ADP we observed here. Other candidate mechanisms are inhibition of slow K^+^ currents, including Ca^2+^-activated K^+^ currents, which have been reported in hippocampal neurons [26]–[28],[35],[41]–[43], and activation of exchanger currents [1]. These effects may also contribute somewhat to the medium ADP, as DHPG induced a small medium ADP even at very negative holding potentials, where activation of VGCCs is limited. At normal (i.e., resting) membrane potentials, inhibition of K^+^ currents could enhance the contribution of VGCCs.

If activation of R-type Ca^2+^ channels were the only requirement for the medium ADP, we would not expect its inhibition by chelating intracellular Ca^2+^ or interfering with Ca^2+^ release from internal stores. Indeed, either of these findings could be presented in support of a Ca^2+^-activated cation current as the primary mechanism. However, it is possible that intracellular Ca^2+^ elevation is required for modulation of the VGCCs following activation of mGluRs by DHPG. Indeed, the pharmacology suggests that the medium ADP requires activation of mGluR5 (and to a lesser extent mGluR1) and activation of G proteins (likely Gq) and PLC. This pathway can lead to several other signal transduction events, including Ca^2+^ release via activation of IP_3_ receptors. Several previous studies have shown that activation of group I mGluRs triggers Ca^2+^ release from internal stores in hippocampal neurons [26],[36],[44]–[48]. In most cases the IP_3_ receptor is the primary mediator of Ca^2+^ release; however, our finding that the medium ADP is inhibited by heparin and ruthenium red suggests that both IP_3_ receptors and Ca^2+^-induced Ca^2+^ release via ryanodine receptors are involved (Figure S8D) [49]. The consequences of this Ca^2+^ release are unknown, but we postulate it is an essential step in the complex cascade of signal transduction events that ultimately result in modulation of the Ca_v_2.3 subunits responsible for the medium ADP.

Our finding that both mGluR5 and mGluR1 activation are required for the full effect of DHPG is consistent with previous work demonstrating the effects of both receptor subtypes in CA1 pyramidal neurons [26],[41],[44], despite greater expression of the mGluR5 subunit [50]–[55]. PLC-dependent and PLC-independent effects have been reported [10],[16],[18], and tyrosine phosphatases have also been implicated in mediating the effects of group I mGluR activation [17]. Clearly, more work is required to elucidate all of the pathways involved in activation of the medium ADP. Even more work will be needed to uncover the transduction mechanisms of the slow ADP. One possible mechanism is a current similar to the inward current mediated by mGluR1 activation previously described in CA3 pyramidal neurons, which was not dependent on G-protein activation (like the slow ADP reported here) but required activation of a Src-family tyrosine kinase [39].

Although bath application of DHPG produced a gradual onset of the medium ADP, it appeared more rapidly (<3 s) when DHPG was applied by pressure application. This suggests that the signal transduction pathways can be activated and reversed very rapidly, an observation that has been used to suggest a membrane delimited mechanism [1]. The block of the medium ADP by chelating intracellular Ca^2+^ or interfering with Ca^2+^ release suggests, however, that membrane delimited signaling alone may be insufficient.

The lack of effect of DHPG when applied to the apical dendrites should be interpreted with caution. Although it is tempting to conclude that the relevant mGluRs may have a perisomatic location, it is also possible that direct activation of the dendrites (e.g., synaptic activation or dendritic Ca^2+^ spikes) could lead to a medium ADP when dendritic mGluRs are activated. More work is needed to determine the distribution of mGluR1 and mGluR5 in CA1 neurons and their physiological effects when activated in various cellular compartments.

An intriguing aspect of the medium ADP is its activity dependence. It was markedly enhanced when pairs of bursts were delivered at intervals of less than 1 s but suppressed during pairs of bursts at longer intervals or when triplets of bursts were delivered to the neurons. Similarly, while short bursts of action potentials produced an ADP, longer trains of spikes did not produce an ADP. The molecular steps responsible for these activity-dependent effects are unknown, but the data using pairs and triplets of bursts were well described by a model consisting of a short-lasting facilitation of unactivated channels and a longer-lasting inhibition of a fraction of all of the channels, independent of activation. These interesting properties may offer a clue to identification of the underlying currents in future voltage-clamp experiments.

Identifying the contribution of mGluR activation to neuronal excitability in vivo will be a crucial step for ultimately establishing the importance of this mechanism for hippocampal function. Accomplishing this task will require that the balance of two competing factors be determined: the enhanced activation of mGluRs during periods of high activity and the activity-dependent inhibition of the ADP during high-frequency spiking. In general, identifying the underlying conductances, their possible molecular composition, and the signal-transduction steps and molecular players involved in their activation and modulation will be critical for determining how excitability is regulated via changes in the AHP/ADP in vivo. This knowledge would facilitate the use of molecular genetics to study the effects of these mechanisms on hippocampal function in vivo with single-unit recordings and behavioral analysis.

## Materials and Methods

Slice experiments were performed in the USA and approved by the Northwestern University Animal Care and Use Committee. Oocyte experiments were performed in Korea and approved by the Sogang University Animal Care Committee.

### Hippocampal Slices

Hippocampal slices were prepared from male Wistar rats 25–45 d old or mice (C57BL/6J or 129S1/SvImJ or Ca_v_2.3 knockout) 21–35 d old. Voltage-clamp experiments were done on slices prepared from younger rats (13–18 d old) in order to reduce space-clamp problems. Knockout mice were derived from 129S1/SvImJ (brown mouse) embryonic stem-cell injections in C57BL/6J (black) mice [29]. The founder mice were bred to C57BL/6J females; therefore, offspring of the knockout mice had either light brown, dark brown, or black coat color.

Following anesthesia with halothane or isoflurane, animals were perfused through the heart with ice-cold ACSF (see below). The brain was removed rapidly and mounted in a near-horizontal plane for preparation of 300 µm hippocampal slices using a vibratome. Slices were prepared in either ice-cold ACSF or sucrose-based solution, then transferred to a chamber containing oxygenated ACSF (no sucrose) at approximately 35°C for half an hour. The slice chamber was then maintained at room temperature and slices were removed individually for electrophysiological recordings.

### Slice Electrophysiology

Whole-cell current-clamp recordings were obtained at 33±2°C. Patch-clamp electrodes were pulled from 2.0 mm outer diameter borosilicate glass and filled with a K-gluconate-based intracellular solution (see below). Electrode resistance was 3–6 MΩ in the bath and series resistance was 5–20 MΩ during the recordings. Current-clamp recordings were obtained with Dagan BVC-700 amplifiers, using appropriate bridge balance and electrode-capacitance compensation. Voltage-clamp recordings with appropriate capacitance and series resistance compensation were performed at room temperature (23–25°C) and monitored with an Axopatch 200B amplifier (Molecular Devices, Union City, CA). Data acquisition and analysis was performed using custom software written for Igor Pro. Statistical tests included the paired or unpaired *t* test and analysis of variance (one-way ANOVA or repeated measures one-way ANOVA) with Tukey's post hoc comparisons. All statistical analyses were performed using Prism 4 software and in most cases detailed results are provided in the figure legends.

For the hyperpolarizations shown in Figure 6, the −6 nA, 2 ms current steps produced hyperpolarizations that briefly (1–8 ms) exceeded the −10 V limit of the analog-to-digital converter. The peak hyperpolarization was therefore estimated by extrapolating double-exponential fits of the response to the peak time predicted from linear fits of the rising phase. The estimated peaks were −119±3 mV for the early steps (*n* = 12) and −117±2 mV for the late steps (*n* = 11). The small clipping effect had an insignificant effect on the subtraction procedure used (see Results), because the effect is measured when the hyperpolarization had decayed back to rest, more than 40 ms after the clipping ended.

### Synaptic Stimulation

To test whether synaptic activation can induce the post-burst ADP, 5 brief action potentials were somatically injected either with or without synaptic stimulation and the responses were monitored once every 5 min with 1 min delay between two conditions. In both the MPEP/LY and control groups, experiments were performed in the presence of blockers of ionotropic glutamate receptors (30 µM CNQX and 50 µM D-AP5) and GABA receptors (2 µM SR95531 and 1 µM CGP55845). Bipolar borosilicate theta glass stimulation electrodes (Sutter Instruments) filled with ACSF were used in conjunction with Dagan BSI-950 biphasic stimulus isolator. Stimulating electrodes were placed in proximal stratum radiatum and at least 100 µm away from the recorded cell and toward CA3. Stimulus intensity was set to produce a 5–11 mV ADP during synaptic stimulation.

### Slice Solutions and Drugs

Normal ACSF had the following composition (mM): 125 NaCl, 2.5 KCl, 25 NaHCO_3_, 1.25 NaH_2_PO_4_, 1 MgCl_2_, 2 CaCl_2_, 25 Dextrose (Fisher Scientific; Sigma). In some cases slices were prepared in a modified ACSF in which 125 mM NaCl was replaced with 75 mM NaCl and 75 mM sucrose. In many experiments, drugs were added to the bath (see below). The bath perfusion rate was 2–3 ml/min.

The intracellular recording solution had the following composition (mM): 115 K-gluconate, 20 KCl, 10 Na_2_phosphocreatine, 10 HEPES, 2 MgATP, 0.3 NaGTP, 0.1% Biocytin (Fisher Scientific; Sigma). In some experiments, drugs were added to the intracellular solution (BAPTA, GDP-β-S, U73122, U73343, ruthenium red, and heparin; see below); for BAPTA-containing internal solution, the K-gluconate concentration was reduced to 100 mM. The K-gluconate based internal solution was used because the properties of CA1 pyramidal neurons are more stable with this solution than with K-Methylsulfate based solutions [56].

For voltage-clamp experiments in slices, patch electrodes (3–6 MΩ in bath) were filled with intracellular solution containing the following (in mM): 110 Cs-gluconate, 25 TEA-Cl, 10 HEPES, 2 EGTA, 4 Mg-ATP, and 0.5 Na-GTP, 5 Na_2_-phosphocreatine, pH 7.3 with CsOH. R- and T-type calcium currents were isolated pharmacologically by preincubating the slices in a mixture containing ω-conotoxin MVIIC (2 µM), ω-conotoxin-GVIA (2 µM), and ω-agatoxin IVA (0.2 µM) to block N-, P-, and Q-type Ca^2+^ currents and cytochrome *c* (0.1 mg/ml to block nonspecific toxin binding) for>1 h at room temperature. Nifedipine (20 µM) and TTX (1 µM) were bath applied to block L-type Ca^2+^ currents and Na^+^ currents, respectively. Recordings were performed in modified ACSF solution containing the following (in mM): 125 NaCl, 25 NaHCO_3_, 2.5 KCl, 1.0 MgCl_2_, 2.0 CaCl_2_, 1.25 NaH_2_PO_4_, 25 NaHCO_3_, and 10 dextrose, 2 CsCl, 5 4-AP, 10 TEA-Cl, pH 7.4.

The following drugs were obtained from Tocris: (S)-3,5-Dihydroxyphenylglycine (DHPG), (S)-(+)-a-Amino-4-carboxy-2-methylbenzeneacetic acid (LY367385), 2-Methyl-6-(phenylethynyl)pyridine hydrochloride (MPEP), Ammoniated ruthenium oxychloride (Ruthenium Red), 1,4-Dihydro-2,6-dimethyl-4-(3-nitrophenyl)-3,5-pyridine dicarboxylic acid 2-methyloxyethyl 1-methylethyl ester (Nimodipine), (6aR,11aS,11bR)-rel-10-Acetyl-2,6,6a,7,11a,11b-hexahydr o-7,7-dimethyl-9H-pyrrolo[1′,2′:2,3]isoindolo[4,5,6-cd] indol-9-one (CPA), D-(−)-2-Amino-5-phosphonopentanoic acid (D-AP5), 6-Cyano-7-nitroquinoxaline-2,3-dione disodium (CNQX disodium salt), (2S)-3-[[(1S)-1-(3,4-Dichlorophenyl)ethyl]amino-2-hydro xypropyl](phenylmethyl)phosphinic acid hydrochloride (CGP 55845 hydrochloride), and Octahydro-12-(hydroxymethyl)-2-imino-5,9∶7,10a-dimethan o-10aH-[1],[3]dioxocino[6,5-d]pyrimidine-4,7,10,11,12-pen tol citrate (Tetrodotoxin citrate).

The following drugs were obtained from Sigma: 1,2-Bis(2-aminophenoxy)ethane-N,N,N',N'-tetraacetic acid tetrapotassium salt (BAPTA), 1-[6-[((17β)-3-Methoxyestra-1,3,5[10]-trien-17-yl)amino]hexyl]-1H-pyrrole-2,5-dione (U73122), 1-[6-[((17β)-3-Methoxyestra-1,3,5[10]-trien-17-yl)amino]hexyl]-2,5-pyrrolidinedione (Uf73343), Guanosine 5′-[β-thio]diphosphate trilithium salt (GDP-β-S), Heparin sodium salt (from porcine intestinal mucosa average mol wt ∼3,000 kD), Nickel(II) chloride hexahydrate (NiCl_2_), 2-(3-Carboxypropyl)-3-amino-6-(4 methoxyphenyl)pyridazinium bromide (SR 95531), Dextrose, K-gluconate, sodium phosphocreatine, HEPES, MgATP, NaGTP, and biocytin.

In most experiments, DHPG was applied by bath perfusion at a concentration of 4 µM. Some batches of DHPG were more potent than others, so in some cases it was necessary to reduce the DHPG concentration to as low as 2 µM in order to prevent additional spiking following the triggered burst of action potentials. Experiments using 2–4 µM DHPG were pooled together for analysis. In most experiments, the membrane potential was held at −65 mV, which required very small current injections (< 50 pA). DHPG application resulted in a depolarization of 3–5 mV, so the holding potential was adjusted to −65 mV with hyperpolarizing holding current. In some experiments (as noted), DHPG (500 µM DHPG) or ACSF was applied to the cell via pressure application from a broken patch pipette. Pressure (10 psi, 0.2 s) was applied via a Dagan PMI-100 pressure micro-injector. Current was injected to the cell within 3 s of pressure application. For experiments with mouse slices, bath application was performed using 10 µM DHPG, yielding an ADP similar to that observed with 4 µM DHPG in rat slices.

### Oocyte Electrophysiology

The cDNAs for the Ca_v_3.2 (accession number AF051946), Ca_v_2.3 (L27745), β_3_ (M88751), α_2_δ_1_ (M86621), and mGluR5 (D10891) were subcloned into a high expression vector pGEMHEA, which contains the 5′ and 3′ untranslated regions of the *Xenopus* β globin gene, linearized, and transcribed into cRNA using T7 RNA polymerase according to the manufacturer's protocol (Ambion, Austin, TX, USA).

Oocytes were obtained from female *Xenopus laevis* (Nasco, WI, USA) using a standard procedure. Several ovary lobes were surgically removed from mature female *Xenopus laevis* and torn into small clusters in SOS solution (in mM: 100 NaCl, 2 KCl, 1.8 CaCl_2_, 1 MgCl_2_, 5 HEPES, 2.5 pyruvic acid, 50 µg/ml gentamicin, pH 7.6). The follicular membranes were removed by digestion in Ca^2+^-free Barth's solution (in mM: 88 NaCl, 1 KCl, 2.4 NaHCO_3_, 0.82 MgSO_4_, pH 7.4) containing 20 mg/ml collagenase (SERVA, Heidelberg, Germany). Oocytes were injected under a stereo-microscope with 2–20 ng of cRNA using a Drummond Nanoject pipette injector (Parkway, PA, USA) attached to a Narishige micromanipulator (Tokyo, Japan).

Barium currents were measured at room temperature 4 to 8 d after cRNA injection using a two-electrode voltage-clamp amplifier (OC-725C, Warner Instruments, Hamden, CT, USA). Microelectrodes (Warner Instruments, Hamden, CT, USA) were filled with 3 M KCl and their resistances were 0.2–1.0 MΩ. The 10 mM Ba^2+^ bath solution contained (in mM): 10 Ba(OH)_2_, 90 NaOH, 1 KOH, 5 HEPES (pH 7.4 with methanesulfonic acid). The currents were sampled at 5 kHz and low pass filtered at 1 kHz using the pClamp system (Digidata 1322A and pClamp 8; Axon instruments, Foster City, CA, USA). Peak currents and exponential fits to currents were analyzed using Clampfit software (Axon instruments, Foster City, CA, USA).

## Supporting Information

Figure S1 **mGluR agonist quisqualate induce the post-burst ADP.** (A) Response to five brief current injections (2 nA, 2 ms each) to evoke a burst of action potential in control (t = 0 min) and 0.5 µM quisqualate (t = 15 min). (B) Time course of the post-burst potential following application of 0.5 µM quisqualate. For comparison, the time course of the post-burst potential in DHPG (Figure 2) is also shown. (C) Δ Post-burst potential in control (t = 0 min) and agonist (DHPG or quisqualate; t = 15 min). (D) Change in post-burst potential in DHPG and quisqualate (*n* = 8 cells). No significant differences were observed between DHPG and quisqualate.
(1.61 MB EPS)

Figure S2 **Time course of DHPG effects.** (A) Perfusion of ACSF containing 2–4 µM DHPG resulted in a gradual transition from a post-burst AHP to a post-burst ADP. Washout of DHPG resulted in reversal of the effect. Example responses are shown for control (black), DHPG (red), and washout (blue). Stimulus is five brief current stimuli (as in Figure 1E) and the time interval between trials is 20 s. Action potentials are truncated. (B) Summary of the time course of DHPG and washout effects and the change in post-burst potential at t  =  15 min (*n*  =  8 cells). Paired *t* test, ** *p* < 0.001.
(2.48 MB EPS)

Figure S3 **The post-burst potential depends on the number and frequency of action potentials.** (A) In normal ACSF (control) the post-burst AHP increased with the number of action potentials. Stimulus is 1, 3, 5, or 10 brief current stimuli and the time interval between trials is 20 s. (B) Perfusion of 2–4 µM DHPG resulted in gradual conversion of the post-burst AHP to an ADP. The size of the ADP was dependent on the number of action potentials. (C) Examples of responses from the experiments shown in (A) and (B). Action potentials are truncated. In each pair of traces, the gray traces were obtained at t  =  0 min and the colored traces were obtained at t  =  15 min. Arrows indicate the amplitude (post-burst potential) or the difference between the AHP and the ADP (Δ post-burst potential; t  =  15 min minus t  =  0 min response), measured at the time of the AHP peak in control (top to bottom: 78, 68, 58, 58 ms after the last action potential). (D) Δ post-burst potential as a function of the number of action potentials in control and DHPG. Repeated-measures one-way ANOVA, *p*  =  0.65 for control, *p* < 0.0001 for DHPG; post hoc tests: versus 1 AP, * *p* < 0.01, ** *p* < 0.001; versus 3 APs, # *p* < 0.01, ## *p* < 0.001. (E) Δ post-burst potential in DHPG as a function of the action potential frequency (5 spikes; not the same group of cells as in D). Repeated-measures one-way ANOVA, *p* < 0.0001; Tukey’s post hoc tests: versus 20 Hz, ** *p* < 0.001; 100 versus 50 Hz, *p* > 0.05.
(10.14 MB EPS)

Figure S4 **Time course of the post-burst ADP and AHP in response to somatic action potentials paired with or without synaptic stimulation**. (A and C) Time course of the post-burst ADP and AHP. (B and D) Normalized change in the post-burst ADP and AHP. Control (A and B), MPEP/LY (C and D), *n* = 5 in each condition.
(1.47 MB EPS)

Figure S5 **Longer trains of action potentials attenuate the post-burst ADP.** (A) Typical responses to bursts of 5, 20, or 50 action potentials obtained from single cell in control (black) or following application of 2 µM DHPG (red). Expansion of the dotted box on the left is shown on the right. The dotted lines indicate the baseline membrane potentials. (B) Post-burst potentials in control and DHPG measured at the time of the AHP peak in control as a function of the number of action potentials. (C) Δ post-burst potential as a function of the number of action potentials. Repeated-measures one-way ANOVA, *p*<0.0001; Tukey's post hoc tests: 5 versus 50 APs, ** *p*<0.01; 5 versus 20 APs, *p*>0.05 (*n* = 10 cells).
(2.30 MB EPS)

Figure S6 **Effects of BAPTA and CPA on the DHPG-induced medium ADP.** (A) Sample traces of the effect of DHPG in the presence of BAPTA (10 mM intracellular) or CPA (20 µM bath incubation). Black traces are control, red DHPG (BAPTA), and blue DHPG (CPA). (B) Time course of effects of DHPG in control (black), BAPTA (red), and CPA (blue). (C) Summary of the effects of DHPG at t = 15 min following application of DHPG. One-way ANOVA, *p*<0.0001, with post hoc tests versus control: ** *p*<0.001.
(1.86 MB EPS)

Figure S7 **Effects of DHPG on the medium ADP in two strains of wild-type (WT) mice and different colored littermates in Ca_v_2.3 knockouts.** (A) C57BL/6J WT. (B) 129S1/SvImJ WT. (C) Ca_v_2.3 KO, black. (D) Ca_v_2.3 KO, light brown. (E) Ca_v_2.3 KO, dark brown. (F) Summary of the change in post-burst potential t = 15 min after application of 10 µM DHPG for all groups. One-way ANOVA of data at t = 15 min after application of DHPG, *p*<0.001; Tukey's post hoc tests: versus C57BL/6J, ** *p*<0.001. No significant differences were observed between wild-type strains or between knockout groups. For panels A–E, the interval between traces is 20 s.
(1.07 MB EPS)

Figure S8 **Signal transduction pathways of the medium and slow ADP.** (A) Example traces for Control (DHPG) and Nickel (DHPG, 50 µM NiCl_2_) effects on the medium and slow ADP. Application of nickel inhibited the medium ADP, but not the slow ADP. Experiments were performed as shown in Figure 5B. Traces are shown in normal ACSF (gray, t = 0 min) and 2.5 µM DHPG (t = 12 min, red; t = 25 min, black in control or green in Nickel). The medium ADP amplitude was measured 58 ms after the last spike; slow ADP amplitude was measured 2 s after the last spike (arrow). Stimulus is five brief current injections and action potentials are truncated. (B) Summary of experiments with bath application of nimodipine (Nim, 10 µM) and nickel (Ni, 50 µM). Data are normalized to the ratio of DHPG (t = 25 min) to DHPG (t = 12 min) in normal ACSF with 2–4 µM DHPG. ANOVA for medium ADP, *p*<0.0001, with Tukey's post hoc tests versus control: ** *p*<0.001. ANOVA for slow ADP, *p* = 0.79. (C) Example traces for signal transduction experiments with drugs applied intracellularly (BAPTA) or pre-incubation (15 min) in the bath (CPA). Responses in normal ACSF (gray, t = 0 min) and after 15 min in 2–4 µM DHPG with intracellular BAPTA or pre-incubation in CPA. BAPTA blocked the medium ADP and slow ADP; CPA blocked the medium ADP, but not the slow ADP. Stimulus is five brief current injections and action potentials are truncated. (D) Summary of effects of signal transduction drugs on the DHPG-induced medium ADP and slow ADP. One-way ANOVA, *p*<0.0001 for both medium ADP and slow ADP, with Tukey's post hoc tests versus control: * *p*<0.01. Drug concentrations: 10 µM MPEP, 25 µM LY367385 (LY), 10 mM BAPTA, 20 µM CPA, 200 µM ruthenium red (RR), 1 mg/ml heparin, 200 µM GDP-β-S, 5 µM U73122, 5 µM U73343.
(4.71 MB EPS)

Figure S9 **Modeling activity dependence of the medium ADP.** (A) Model 1 includes a short-lasting facilitation of activation probability and longer-lasting inhibition of the activated fraction of the current (I_ADP_) responsible for the medium ADP. The ratio of I_ADP_ for the second burst to the first burst is plotted for different intervals. Experimental data are plotted (as in Figure 10; two-burst data in blue, three-burst data in red), with dotted fit lines from the model. (B) Model 2 includes a short-lasting facilitation of activation probability and longer-lasting inhibition that is uncoupled from activation. The ratio of I_ADP_ for the second burst to the first burst is plotted for different intervals. Experimental data (as in Figure 10) are plotted in color, with dotted fit lines from the model.
(0.88 MB EPS)

Text S1 **Description of medium ADP modeling.**
(0.08 MB DOC)

## Abbreviations

ACSF: artificial cerebrospinal fluid
ADP: afterdepolarization
AHP: afterhyperpolarization
CPA: cyclopiazonic acid
mGluRs: metabotropic glutamate receptors
PLC: phospholipase C
TTX: tetrodotoxin citrate
VGCCs: voltage-gated calcium channels
