# Supplementary material for: A Post-Burst Afterdepolarization Is Mediated by Group I Metabotropic Glutamate Receptor-Dependent Upregulation of Cav2.3 R-Type Calcium Channels in CA1 Pyramidal Neurons
Source: PLoS Biol. 2010 Nov 16;8(11):e1000534. doi: 10.1371/journal.pbio.1000534 (PMC2982802; doi:10.1371/journal.pbio.1000534)
Supplement: Text S1 — Description of medium ADP modeling. (0.08 MB DOC) [file pbio.1000534.s010.doc]

**Text S1.** Description of medium ADP modeling.

The medium ADP data from pairs and triplets of bursts (Figure 10) were fit using two different models (Figure S9). Both models were coded using a system of equations in Igor Pro.

*Features common to both models*

A key feature of both models is that a finite current (I_ADP_) is available to contribute to the medium ADP, which is activated by a burst with a finite probability (P_act_). The first burst activates about one-third of I_ADP_. The second burst activates almost two-thirds of I_ADP_ at short intervals (<0.35 sec), due to a short-lasting facilitation of P_act_. The facilitation factor can be large; it increases P_act_ additively, but P_act_ cannot exceed 1.0. The facilitation factor decays with a time constant of 0.25 sec. Thus, in both models, P_act_ is close to 1.0 for about 0.25 sec after the first burst, accounting for the flat portion of the two-burst curve at intervals of 0.1 and 0.2 sec (Figure S9, blue).

Also common to both models is an inhibitory factor that inhibits about one-third of the available I_ADP_ immediately following a single burst and decays with a time constant of 10-15 seconds. Thus, although P_act_≈1 for short intervals, only about two-thirds of I_ADP_ is available at this time, yielding a ratio (ADP_2_/ ADP_1_) of almost two.

*Differences between the models*

The major difference between the models is in the inhibitory process. In Model-1, the fraction of I_ADP_ that is inhibited is the same fraction that is activated, as expected for conventional inactivation of voltage-gated channels. A consequence of this model, however, is that following a pair of bursts, all of I_ADP_ is inhibited (one-third from the first burst and almost two-thirds from the second burst), so almost none is available on the third burst. This is inconsistent with the results of our three-burst experiments (Figure 10B and Figure S9A).

To solve this problem, in Model-2 the inhibition process is uncoupled from activation and instead is applied to a fraction of the total I_ADP_. This is distinctly different from inactivation of voltage-gated channels, but it can be thought of, for example, as a global Ca^2+^-meditated inhibitory process that acts on all available channels. In this model, a single burst inhibits about one-third and a pair of bursts inhibits almost two-thirds of the total I_ADP_ (Figure S9B). Implicit in this model is the assumption that if VGCCs are responsible for the medium ADP (and hence I_ADP_) any inhibition that is coupled to activation of the current must recover quickly enough to be available for activation by the second burst at an interval of 0.2 seconds.

*Mathematical description of the model*

Both models are based on a total pool of channels (C_tot_), of which some are available to be activated (C_avl_) and some are inhibited (C_inh_). Thus,

C_avl_ = C_tot_ – C_inh_

For simplicity, C_tot_ is normalized to unity. Thus,

C_avl_ = 1 – C_inh_

The fraction of available channels that is activated (C_act_) by a burst of action potentials is determined by a finite probability of activation (P_act_).

Thus, C_act_ = C_avl_ * P_act_

The fraction of the channels that is inhibited is determined by a finite probability of inhibition (P_inh_), which decays exponentially according to a time constant (τ_inh_). Inhibition operates in very different ways in the two models. In the first model, only activated channels can be inhibited; in the second model, any available channels (i.e. those not already inhibited) can be inhibited. Thus for a pair of bursts at any time interval (t), the pool of channels inhibited by the first burst and still inhibited at the time of arrival of the second burst is computed as follows:

For model 1: C_inh_’ = C_act_ * e^-t/τinh^

For model 2: C_inh_’ = C_avl_ * P_inh_ * e^-t/τinh^

In addition, the probability of activation (P_act_) is increased by prior activity (i.e. the first burst) through facilitation. This increases P_act_ by a finite amount (P_fac_), which decays exponentially according to a time constant (τ_fac_). Thus, for a pair of bursts, the activation probability for the second burst can be computed as follows:

P_act_’ = P_act_ + P_fac_ * e^-t/τfac^ ,

with the limitation that the new activation probability cannot exceed one.

The fraction of channels activated is computed as follows (for both models):

C_act_’ = (1 – C_inh_’) * P_act_’

The dashed blue lines in Figure S9 were computed by performing these calculations and plotting C_act_’/C_act_ as a function of a time interval.

To compute the response to a third pulse, values were computed as described above for the first time interval (0.1 sec), the initial values were replaced with the new values (e.g. P_act_ = P_act_’), and new values were again computed for a range of intervals (using t=t+0.1).

The following constants were used in the computations:

|  | **Model 1** | **Model 2** |
| --- | --- | --- |
| P_act_ | 0.35 | 0.36 |
| P_inh_ | 1.0 | 0.35, 0.25 |
| τ_inh_ | 15 sec | 10 sec |
| P_fac_ | 1.50 | 1.50, 1.05 |
| τ_fac_ | 0.29 sec | 0.25, 0.30 sec |

These values were found to give good fits to the experimental data, as shown, except for the three-burst experiment using model 1. Where two values are noted in a single table cell, it was necessary to adjust these values for computation of the three-burst results in order to achieve optimal fits of the data.

Some qualitative features of the data are worth noting:

1) At long intervals, inhibition dominates, with a minimum activation ratio of around 0.7 at an interval of 2 seconds (two-burst experiment). This implies that the first burst of action potentials inhibits at least 30% of the channels for longer than a second.

2) At short intervals in the two-burst experiments, facilitation dominates, suggesting that it is a strong process, but it operates on a shorter time scale than inhibition.

3) The similar ratio for intervals of 0.1 and 0.2 seconds suggests that the facilitation process saturates the probability of channel activation at one for these short intervals. At these intervals, the medium ADP ratio of 1.8 suggests that the maximum initial P_act_ is approximately 0.36, as larger values would necessarily result in lower ratios, especially given that a large fraction of channels is inibited by each burst (point #1).

4) If P_act_ is high (point #3) and inhibition is high (point #1) and only activated channels can be inhibited (model 1) then almost all activated channels must be inhibited. Thus, for model 1, the two-burst experiment leads to activation and inhibition of almost all channels and thus a very small ADP following a third burst of action potentials. This combination of observations renders model 1 implausible.

5) The fact that ratios in the three-burst experiment are always smaller than in the two-burst experiment, but always greater than zero and never greater than one, suggests that the fraction of channels inhibited by each burst of action potentials is large and cumulative, but not complete.

These features of the data were helpful in arriving at parameters that provided the best fits of the data. Code for the model was written using Igor Pro (Wavemetrics) and will be provided on request (spruston@northwestern.edu).
